# Special Issue “Nephrotic Syndrome: Pathomechanism, Diagnostics, and Novel Treatment Options”

**DOI:** 10.3390/biomedicines12122862

**Published:** 2024-12-17

**Authors:** Kan Katayama, Ryosuke Saiki, Kaoru Dohi

**Affiliations:** Department of Cardiology and Nephrology, Mie University Graduate School of Medicine, Tsu 514-8507, Japan; ryosuke-s@med.mie-u.ac.jp (R.S.); dohik@med.mie-u.ac.jp (K.D.)

Nephrotic syndrome (NS) is characterized by massive proteinuria, hypoproteinemia, and edema [1] and can be divided into steroid-sensitive and steroid-resistant NS, often leading to end-stage renal failure [2]. Approximately 30% of childhood-onset focal segmental glomerulosclerosis (FSGS) have been found to be hereditary FSGS due to podocyte-related genes [3], and approximately 5–10% of adult-onset FSGS have also been found to be hereditary [4]. The aim of this Special Issue is to deepen our knowledge by gathering original research articles and review articles focused on NS.

Kidney failure significantly increases mortality in multiple myeloma (MM), with cast nephropathy (CN) being a particularly severe complication. Klank D et al. examined the urine light chain (LCurine)/estimated glomerular filtration rate (eGFR) quotient to rule out CN in myeloma-associated kidney failure. The study validated the LCurine/eGFR ratio as a noninvasive diagnostic tool for CN, demonstrating 100% sensitivity and 85.7% specificity in identifying high-risk patients without requiring a kidney biopsy.

Nephrotic proteinuria (NP) and NS are common and severe complications after kidney transplantation, significantly associated with poor graft survival. Ortega MJ et al. found that early-onset NS (within 3 months of NP) markedly increased the risk of graft loss, emphasizing the critical impact of timing on outcomes.

Lee CL et al. compared intradialytic exercise (IDE) with home-based exercise (HBE) in hemodialysis (HD) patients. The rising prevalence of end-stage kidney disease has increased the number of patients on HD, which is often associated with adverse effects like muscle loss and reduced quality of life. IDE and HBE have shown promise in improving physical function, health-related quality of life (HRQoL), and cardiopulmonary health in HD patients, but challenges like adherence and implementation feasibility persist, warranting further research to optimize protocols and assess long-term benefits.

Wendt R et al. reviewed diseases associated with NS. Recent advances in understanding primary glomerular diseases and NS have improved disease phenotyping, nephrogenetics, and biomarker use, leading to more accurate diagnoses and tailored treatments while reducing unnecessary immunosuppression. The review highlights progress in various NS-associated conditions, including podocytopathies, membranous nephropathy, and systemic diseases, alongside emerging therapeutic options and updated insights into their management.

Mizdrak M et al. reviewed endocrine disorders in NS. NS is characterized by severe proteinuria, hypoalbuminemia, edema, and hyperlipidemia, leading to significant morbidity and mortality. The review explores the interplay between NS and endocrine disorders, highlighting common complications like hypothyroidism, hormone deficiencies, and vitamin D imbalances, as well as the overlooked kidney effects of endocrine diseases.

Kovalik ME et al. reviewed the paradoxical roles of interleukins in NS. Interleukins, known for their pro-inflammatory effects, also exhibit cytoprotective roles in NS by promoting podocyte survival through pathways like phosphoinositide-3-kinase (PI3K)/AKT. The review emphasizes the dual role of interleukins as both contributors to and mitigators of podocyte injury, suggesting their potential as therapeutic targets for podocytopathy in NS.

Nell D et al. reviewed complement activation in NS. NS, characterized by significant proteinuria, often stems from glomerular basement membrane or podocyte damage, with growing evidence implicating the complement system in its pathogenesis. The review explores complement–glomerular interactions, highlights targeted complement inhibition as a potential therapy, and discusses emerging diagnostic tools and treatments for nephrotic glomerular diseases.

Saiki R et al. reviewed recent advances in proteinuric kidney disease/NS, especially from knockout/transgenic mouse models. Proteinuria and NS are associated with high mortality, and advances in genetics have identified new causative genes for kidney diseases. Studies using mouse models have deepened the understanding of the genetics and pathophysiology of various kidney diseases, paving the way for future personalized treatments.

This Special Issue includes research papers on noninvasive diagnostic tools in MM-associated renal disorders and the consequences of NP and NS after renal transplantation. Review articles include a comparison of exercise in the dialysis room versus exercise at home in HD patients, an updated review of diseases associated with NS, a review of endocrine disorders in NS, the role of interleukins in NS, complement activation in nephrotic glomerular disease, and proteinuric kidney disease/a deeper understanding of lessons learned from knockout/transgenic mouse models with respect to NS. We thank all the authors for their contributions to this Special Issue and hope that it will lead to further advances in understanding the mechanisms of NS and in the development of new therapeutic agents.
